# Season and temperature do not affect cumulative live birth rate and time to live birth in *in vitro* fertilization

**DOI:** 10.3389/fendo.2023.1156299

**Published:** 2023-06-21

**Authors:** Mingze Du, Junwei Zhang, Zhancai Wei, Li Li, Xinmi Liu, Manman Liu, Xingling Wang, Yichun Guan

**Affiliations:** The Reproductive Center, The Third Affiliated Hospital of Zhengzhou University, Zhengzhou, Henan, China

**Keywords:** IVF, season, temperature, cumulative live birth, time to live birth

## Abstract

**Objective:**

To explore whether season and temperature on oocyte retrieval day affect the cumulative live birth rate and time to live birth.

**Methods:**

This was a retrospective cohort study. A total of 14420 oocyte retrieval cycles from October 2015 to September 2019. According to the date of oocyte retrieval, the patients were divided into four groups (Spring(n=3634);Summer(n=4414); Autumn(n=3706); Winter(n=2666)). The primary outcome measures were cumulative live birth rate and time to live birth. The secondary outcome measures included the number of oocytes retrieved, number of 2PN, number of available embryos and number of high-quality embryos.

**Results:**

The number of oocytes retrieved was similar among the groups. Other outcomes, including the number of 2PN (P=0.02), number of available embryos (p=0.04), and number of high-quality embryos (p<0.01) were different among the groups. The quality of embryos in summer was relatively poor. There were no differences between the four groups in terms of cumulative live birth rate (P=0.17) or time to live birth (P=0.08). After adjusting for confounding factors by binary logistic regression, temperature (P=0.80), season (P=0.47) and duration of sunshine(P=0.46) had no effect on cumulative live births. Only maternal age (P<0.01) and basal FSH (P<0.01) had an effect on cumulative live births. Cox regression analysis suggested no effect of season(P=0.18) and temperature(P=0.89) on time to live birth. Maternal age did have an effect on time to live birth (P<0.01).

**Conclusion:**

Although season has an effect on the embryo, there was no evidence that season or temperature affect the cumulative live birth rate or time to live birth. It is not necessary to select a specific season when preparing for IVF.

## Introduction

Climate change poses potential future risks to human health. Climate change may increase the frequency of heat stress, floods, droughts, and severe storms, with adverse effects on human health (1). Earth’s surface temperature is rising as greenhouse gas emissions increase. Earth warmed by approximately 0.85°C between 1880 and 2012 (2). The temperature of the Earth’s surface continues to rise as past emissions remain in the atmosphere and greenhouse gas emissions continue (3). The impact of rising ambient temperature on human health is well known (4). There is growing evidence that maternal heat exposure is associated with an increased risk of stillbirth, preterm birth, low birth weight, placental abruption, low amniotic fluid, and birth defects (5–11). However, little research has been done on fertility in women exposed to heat. Many animal studies have revealed the effects of temperature on fertility. Studies in South Africa on the iconic ostrich (*Struthio camelus*) have found that overheating adversely affects the number of oocytes females lay rather than gamete viability (12). In sows, elevated temperatures can adversely affect the farrowing rate and fertility performance (13–16). In dairy cows, high temperatures can affect embryonic development and endometrial receptivity, thereby reducing cow fecundity (17, 18). Studies on the effect of high temperature on human female fertility suggest that high temperature can adversely affect fertility, but the specific mechanism remains unclear. Barreca et al. showed that exposure to 8-10 months of days with a mean temperature above 80°F causes a significant drop in birth rates (19). A study that analyzed 55 years of data from 65 countries suggested that higher maximum temperatures can negatively affect human fertility and that these effects can persist into the next generation (20). Exposure to higher ambient temperatures is associated with lower ovarian reserve, according to a study from the Massachusetts General Hospital Fertility Center (21).

Natural conception birth rates vary by season (22, 23). Cultural behaviors and sociodemographic influences affect the relationship between fertility and seasons (24, 25). As many factors affecting human reproductive activity are relatively controllable through assisted reproductive technology, many studies have examined assisted reproductive technology as a means of elucidating the relationship between seasonality and reproductive outcomes. However, the results of these studies are controversial. Some studies have suggested that assisted reproductive outcomes are not related to season (26–29). A Belgian study found a link between weather conditions in the month prior to IVF treatment and live birth rates per cycle (30). A University of Arizona study suggested that higher temperatures are associated with higher odds of clinical pregnancy (31). Another recent study found that higher oocyte retrieval day temperatures are associated with higher odds of clinical pregnancy and live birth at the time of frozen embryo transfer cycles, independent of temperature at the time of frozen embryo transfer (32). This suggests that temperature may affect ovarian function more than uterine receptivity.

As global temperatures rise, the effects of temperature on fertility will increase. Several recent studies suggested that higher temperatures may have an impact on IVF clinical pregnancy rates and live birth rates. Should we advise patients to retrieve oocytes at specific temperatures or during certain seasons to improve IVF outcomes? This study aimed to explore whether the temperature on oocyte retrieval day affects the cumulative live birth rate (CLBR) and time to live birth.

## Materials and methods

### Study design and population

This was a retrospective cohort study conducted at the Reproductive Center of the Third Affiliated Hospital of Zhengzhou University. This study was approved by the Ethics Committee of the Third Affiliated Hospital of Zhengzhou University (2022–199–01). The oocyte retrieval time of the included patients was from October 2015 to September 2019. Exclusion criteria were as follows: ① Intracytoplasmic sperm injection (ICSI) or preimplantation genetic testing (PGT) cycle; ② oocyte recipient cycle, oocyte donor cycle, and oocyte freezing recovery cycle; ③ had not obtained oocyte cycle; ④ any chromosome abnormality in either spouse; ⑤ cycle with incomplete data.

According to the date of oocyte retrieval, the patients were divided into four groups (spring, summer, autumn, and winter). Because the meteorological season can better reflect actual climate change, we used the meteorological season as the basis for grouping: spring (March to May); summer (June to August); autumn (September to November), and winter (December to February). Embryo evaluation standards refer to the Istanbul consensus (33). Embryos of grades I, II and III were available embryos, and embryos of grades I and II were high-quality embryos.

The primary outcome measures were CLBR and time to live birth. CLBR: In one IVF cycle (one oocyte retrieval cycle, including fresh embryo transfer and subsequent frozen embryo transfer cycle), the number of cycles of the first live birth (gestation ≥28 weeks, live birth) was used as the numerator, the entire number of oocyte retrieval cycles was used as the denominator, and the end-of-observation standard was to obtain at least one live birth or utilize all of the embryos in the ovulation induction (34). The observation period was 2 years, and single births, twins, or other multiple births were registered as one birth. We used conservative methods to calculate the cumulative live birth rate. The observation and follow-up period was 2 years. Time to live birth: Time from oocyte retrieval to delivery. The secondary outcome measures included the number of oocytes retrieved, number of 2PN, number of available embryos, and number of high-quality embryos.

### Statistical analysis

Zhengzhou’s climate data were obtained from the China Meteorological Administration Meteorological Data Center (http://data.cma.cn/), China’s surface climate data daily value dataset (V3.0). This study extracted the daily average temperature and cumulative daylight duration. All data presented in this article were obtained from the electronic medical record database of The Reproductive Center, The Third Affiliated Hospital of Zhengzhou University.

Quantitative data are presented as the mean ± standard deviation ( $\bar{x}$ ± s), quantitative data were compared using one-way analysis of variance, qualitative data are presented as the percentage (%), and multiple sets of qualitative data were compared using the chi-square test. Binary logistic regression was used to analyze the effects of season and temperature on the clinical outcomes. Factors analyzed included maternal age, basal follicle-stimulating hormone (FSH) level, infertility type, and duration of infertility. Cox regression analysis was used to assess the time to live birth(adjusted by maternal age, basal follicle-stimulating hormone (FSH) level, infertility type, and duration of infertility). When P was <0.05, the difference was considered statistically significant.

All statistical management and analyses were performed using SPSS software, version 22.0.

## Results

### Study population

Overall, 14420 oocyte retrieval cycles from October 2015 to September 2019 were included in the analysis. The cycles were allocated to four groups according to the date of oocyte retrieval: ① spring (March to May); ② summer (June to August); ③ autumn (September to November); ④ and winter (December to February).

### Baseline characteristics

The details of the baseline and cycle characteristics among the four groups are described in **Table 1**. The temperature differed among the groups (spring 18.0 ± 5.9°C; summer, 27.5 ± 2.8°C; autumn, 16.3 ± 6.5°C; and winter, 2.6 ± 3.5°C, P<0.01). There were significant differences in cumulative sunshine at oocyte retrieval (spring 6.6 ± 4.0 h; summer, 6.3 ± 3.9 h; autumn, 4.6 ± 3.8 h; and winter, 3.9 ± 3.3 h, P<0.01). Other basic characteristics, including maternal age (P=0.08), paternal age (P=0.33), infertility type (P=0.20), duration of infertility (P=0.23), body mass index (P=0.34), basal FSH level (P=0.11) and anti-Müllerian hormone (AMH) level (P=0.78) were similar among the groups (**Table 1**).

**Table 1:** Comparison of demographic characteristic among groups (±s).

Characteristic	Spring(n=3634)	Summer(n=4414)	Autumn(n=3706)	Winter(n=2666)	P value
Maternal age (year)	33.0 ± 6.1	32.9 ± 5.8	33.2 ± 5.9	32.9 ± 6.0	0.08
Paternal age (year)	33.8 ± 6.6	33.8 ± 6.4	34.0 ± 6.4	33.9 ± 6.5	0.33
Infertility type					0.20
Primary infertility	1343(37.0%)	1732(39.2%)	1433(38.7%)	1021(38.3%)	
Secondary infertility	2291(63.0%)	2682(60.8%)	2273(61.3%)	1645(61.7%)	
Duration of infertility(year)	3.6 ± 3.0	3.7 ± 3.1	3.7 ± 3.1	3.6 ± 3.1	0.23
Body mass index(Kg/m2)	23.6 ± 3.2	23.6 ± 3.2	23.5 ± 3.2	23.6 ± 3.2	0.34
Basal FSH(IU/L)	7.9 ± 4.1	7.8 ± 4.1	7.8 ± 4.0	7.7 ± 3.8	0.11
AMH(ng/ml)	3.4 ± 4.4	3.5 ± 4.8	3.4 ± 3.5	3.4 ± 3.4	0.78
Temperature(℃)	18.0 ± 5.9	27.5 ± 2.8	16.3 ± 6.5	2.6 ± 3.5	<0.01
Duration of sunshine(h)	6.6 ± 4.0	6.3 ± 3.9	4.6 ± 3.8	3.9 ± 3.3	<0.01

h, hour; AMH, anti-Müllerian hormone; FSH, follicle stimulating hormone.

### Embryo quality by the season of oocyte retrieval

The number of oocytes retrieved was similar among the groups. Other outcomes, including the number of 2PN (P=0.02), number of available embryos (P=0.04), and number of high-quality embryos (P<0.01) differed among the groups. The quality of embryos in summer was relatively poor (**Table 2**).

**Table 2:** Comparison of outcomes of ovarian stimulation (±s).

Characteristic	Spring(n=3634)	Summer(n=4414)	Autumn(n=3706)	Winter(n=2666)	P value
Number of oocytes retrieved	11.3 ± 7.8	11.0 ± 7.1	11.3 ± 7.9	11.4 ± 7.6	0.08
Number of 2PN	7.5 ± 6.0	7.2 ± 5.5	7.4 ± 5.7	7.7 ± 6.0	0.02
Number of available embryos	5.9 ± 5.1	5.6 ± 4.7	5.7 ± 4.9	5.9 ± 4.9	0.04
Number of high-quality embryos	3.4 ± 3.8	3.1 ± 3.4	3.1 ± 3.5	3.4 ± 3.6	<0.01
Cumulative live birth rate	1832(50.4%)	2320(52.6%)	1884(50.8%)	1395(52.3%)	0.17
Time to live birth(d)	324 ± 97	321 ± 94	329 ± 100	323 ± 94	0.08

2PN, 2 pronuclei.

### Clinical outcomes by season and temperature at oocyte retrieval

**Figure 1:**
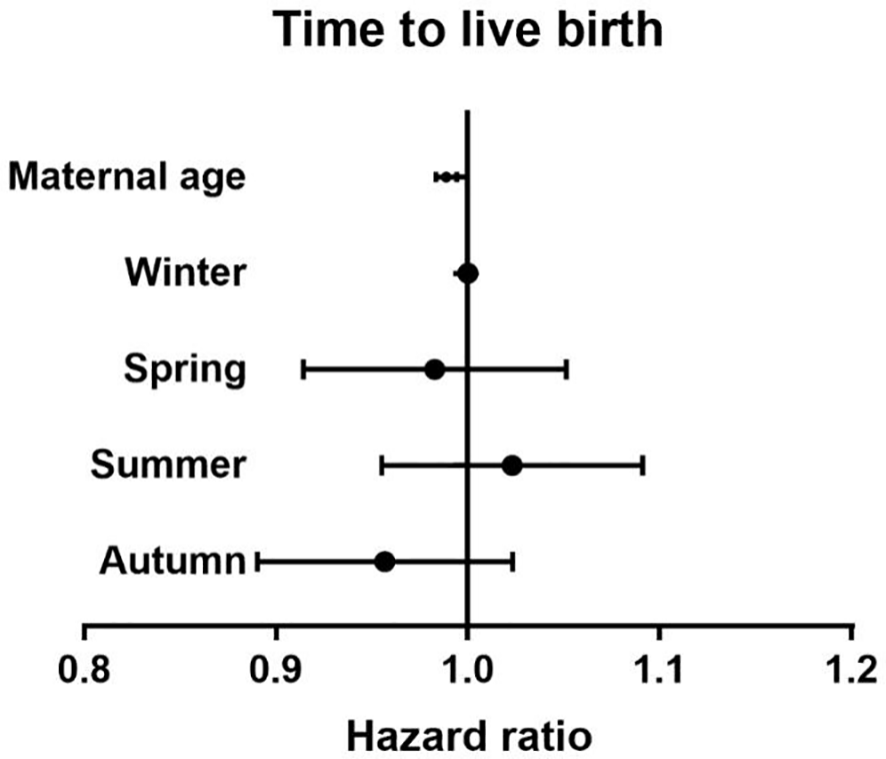
The effect of seasons on time to live birth. Adjust for maternal age, basal follicle-stimulating hormone (FSH), infertility type and duration of infertility.

**Figure 2:**
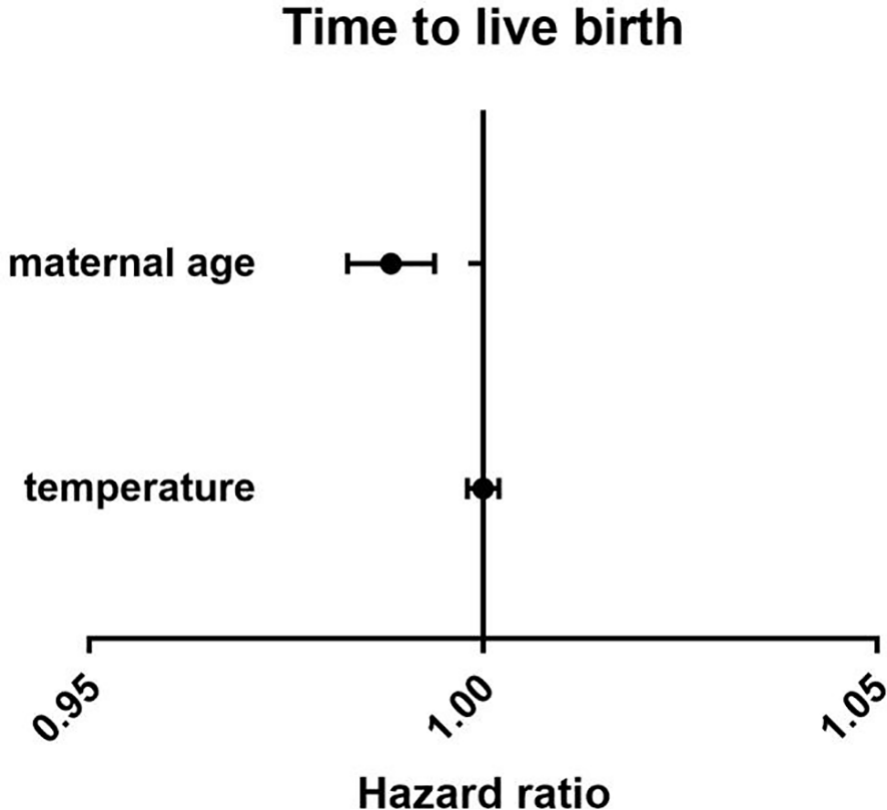
The effect of temperature on time to live birth. Adjust for maternal age, basal follicle-stimulating hormone (FSH), infertility type and duration of infertility.

There were no differences between the four groups in terms of CLBR (P=0.17) or time to live birth (P=0.08). After adjusting for confounding factors by binary logistic regression analysis, season (P=0.47) (**Table 3**), temperature (P=0.80) (**Table 4**) and duration of sunshine (P=0.46) (**Table 5**) had no effect on cumulative live births. Only maternal age (P<0.01) and basal FSH level (P<0.01) had an effect on cumulative live births. Cox regression analysis suggested no effect of season (P=0.18) (**Figure 1**) and temperature (P=0.89) (**Figure 2**) on time to live birth. Maternal age did have an effect on time to live birth (P<0.01).

**Table 3:** The effect of seasons on cumulative live birth rate.

Item	Cumulative live birth rate	
	AOR(95%CI)	P value
Maternal age	0.85(0.84-0.86)	<0.01
Basal FSH	0.92(0.91-0.93)	<0.01
Infertility type	0.95(0.88-1.04)	0.25
Duration of infertility	1.00(0.98-1.01)	0.53
Season		0.47
Winter	1.00(Ref)	
Spring	0.94(0.84-1.05)	
Summer	1.01(0.91-1.12)	
Autumn	0.97(0.87-1.09)	

**Table 4:** The effect of temperature on cumulative live birth rate.

Item	Cumulative live birth rate	
	AOR(95%CI)	P value
Maternal age	0.85(0.84-0.86)	<0.01
Basal FSH	0.92(0.91-0.93)	<0.01
Infertility type	0.95(0.88-1.04)	0.25
Duration of infertility	1.00(0.98-1.01)	0.53
Temperature	1.00(1.00-1.00)	0.80

**Table 5:** The effect of duration of sunshine on cumulative live birth rate.

Item	Cumulative live birth rate	
	AOR(95%CI)	P value
Maternal age	0.85(0.84-0.86)	<0.01
Basal FSH	0.92(0.91-0.93)	<0.01
Infertility type	0.95(0.88-1.04)	0.26
Duration of infertility	1.00(0.98-1.01)	0.53
Duration of sunshine	1.00(0.99-1.01)	0.46

## Discussion

This study explored the effects of temperature and season on the cumulative live birth rate and time to reach live birth. We found that the CLBR and the time to live birth had no relationship with the season or temperature on the oocyte retrieval day.

The effects of season and temperature on human fertility have been studied for many years, but exploring the effects of natural pregnancy also often includes exploring cultural behaviors and sociodemographic factors (24, 25). With the maturity and development of assisted reproductive technology, it has become preferable to explore the effects of season and temperature on human fertility through assisted reproductive technology. Compared with natural pregnancy, assisted reproductive technology provides a good model to study the effects of season and temperature on the female reproductive process. In summer, the number of 2PN, number of available embryos, and number of high-quality embryos were relatively low, consistent with a previous study (35).

Regarding the clinical pregnancy rate and live birth rate, most early studies concluded that season and temperature were not independent factors (28, 36, 37). However, as research has continued, some recent studies suggested a different view. A study from the Chinese University of Hong Kong on IVF treatment suggested that changes in environmental temperature can alter the pregnancy rate (38). The authors of a recent study reported slightly higher clinical pregnancy rates in patients who had oocytes retrieved in June and July, and further analysis found that higher temperatures at the time of oocyte retrieval were associated with higher clinical pregnancy rates but not live birth rates (31). Another study examining frozen embryo transfer cycles reported the interesting finding that summer oocyte retrieval and oocyte retrieval at higher temperatures were associated with higher clinical pregnancy and live birth rates but not with seasonal parameters of frozen transfer day, suggesting that any seasonal effect on IVF success was associated with ovarian function and oocyte quality (32). In a nationwide, register-based cohort study, live birth rates were significantly higher in spring than in summer (39). These findings are controversial, however. This may be due to differences in the meteorological characteristics and geographical environments of the study populations or may be related to inconsistencies regarding time (day of oocyte retrieval/day of embryo transfer) and time length.

To further examine the effects of season and temperature on the day of oocyte retrieval on assisted reproductive technology, we evaluated the CLBR and time to live birth. The cumulative pregnancy rate assessment included the overall treatment outcome of both fresh embryo transfer and subsequent frozen embryo transfer cycles, reflecting the chance of a live birth throughout the course of treatment, which is of greater significance to both the patient and the clinician. CLBR is a more comprehensive and accurate indicator for evaluating the efficacy and safety of treatment. The time to live birth could be used to further evaluate the effectiveness of assisted reproductive technology, and we therefore analyzed the influence of temperature and season on live birth time using Cox regression. The association between seasonal variation and IVF outcomes has not been elucidated. Existing studies have published conflicting reports. Our study also does not support the conclusion that season or temperature affect clinical outcomes. It has been hypothesized that the mechanism by which season may affect the outcome of IVF involves serum vitamin D levels, increased vitamin D synthesis, and increased blood vitamin D levels caused by exposure to more sunlight in summer, which may influence assisted reproductive technology(ART) outcome (40). Evidence as to whether vitamin D levels are associated with IVF outcomes is conflicting, with some studies reporting that increased vitamin D levels improve the likelihood of IVF success (41, 42) and others not supporting this conclusion (43, 44). A 2018 meta-analysis of 11 published cohort studies concluded that adequate vitamin D levels are associated with higher odds of clinical pregnancy and live birth in women undergoing ART (37). However, a recent systematic review and meta-analysis showed that serum vitamin D levels are not associated with IVF/ICSI outcomes (43).

Another strength of the present study is that we selected the cumulative live birth rate and time to live birth as measures of ART outcomes, and we used Cox regression to analyze the time to live birth. Another advantage is that we could adjust for confounding factors such as maternal age, basal FSH level, infertility type, and duration of infertility. In addition, the amount of data in this study was relatively large and reliable.

Of course, there are some limitations to our study. First, we did not consider sperm parameters such as semen concentration, sperm motility, or normal morphology. However, we only included IVF cycles and did not include ICSI cycles with worse semen quality or abnormal fertilization. In addition, we used meteorological data for the day of oocyte retrieval to analyze the outcome, which may also have had an impact on the outcome. And there is a certain difference between the indoor environment and the atmospheric environment. In the future, meteorological data from other time periods may need to be considered to evaluate the outcome, such as the average temperature during oocyte development. Another limitation of the present study is that we did not consider the impact of atmospheric pollutants on the outcome and the effect of temperature on the drug. Finally, this study was a single-center, retrospective analysis, and the results therefore may not be able to be extended to regions with different climatic conditions.

## Conclusion

Although season has an effect on embryos, there was no evidence in the present study that season and temperature affect the cumulative live birth rate and time to live birth. It is therefore not necessary to select a specific season when preparing for IVF. Future multicenter studies should be conducted to further explore this question.

## Data availability statement

The raw data supporting the conclusions of this article will be made available by the authors, without undue reservation.

## Ethics statement

The studies involving human participants were reviewed and approved by Ethics Committee of the Third Affiliated Hospital of Zhengzhou University. Written informed consent for participation was not required for this study in accordance with the national legislation and the institutional requirements.

## Author contributions

MD and YG designed the study and selected the population to be included and excluded. ZW, XL, ML, XW and JZ performed the data extraction and analysis. YG and LL reviewed the data. MD and JZ drafted this article. All authors have approved the final version of the manuscript. All authors contributed to the article and approved the submitted version.
